# 伴MLL-AF9基因重排和肝功能损伤的急性髓系白血病自发缓解一例报告并文献复习

**DOI:** 10.3760/cma.j.issn.0253-2727.2021.10.010

**Published:** 2021-10

**Authors:** 文娟 樊, 婷婷 徐, 丽娜 桑, 洁洁 郭, 亚飞 李, 智信 裴, 中兴 姜

**Affiliations:** 1 郑州大学第一附属医院血液科 450000 Department of Hematology, The First Affiliated Hospital of Zhengzhou University, Zhengzhou 450000, China; 2 河南省人民医院输血科，郑州 50000 Department of Blood Transfusion, Henan Provincial People's Hospital, Zhengzhou 450000, China; 3 焦作市第一人民医院 454002 Jiaozuo First People's Hospital, Jiaozuo 454002, China

**Keywords:** 急性髓系白血病, 自发缓解, MLL-AF9基因重排, 感染, Leukemia, Spontaneous remission, MLL-AF9 rearrangement, Infection

## Abstract

**目的:**

探讨急性髓系白血病（AML）自发缓解的临床特点及可能的机制。

**方法:**

回顾性分析郑州大学第一附属医院诊治的1例伴有MLL-AF9基因重排和肝功能损伤的AML自发缓解患者的临床资料，并进行相关文献报道病例汇总分析。

**结果:**

该患者伴有MLL-AF9基因重排，经历了肺部感染、发热、肝功能损伤，治疗上给予抗感染、输注血制品，未经化疗，患者获完全缓解（CR），随访35个月，仍处于CR，期间伴有轻度间接胆红素升高。1990年至2021年6月文献报道的有骨髓检测支持的AML（非急性早幼粒细胞白血病）自发缓解患者56例，与本次报道的1例汇总分析，共57例患者，其中男37例，女20例，中位年龄51（20～83）岁，中位缓解时间为5个月，52例患者获得CR，5例具有染色体核型资料的长期缓解且至今未复发的病例，3例为正常染色体核型，2例伴有t（9;11）（q21;q23）异常。

**结论:**

AML自发缓解罕见，可能与免疫抑制、基因均有关。

## Abstract (en)

**Objective:**

To explore the clinical features and possible pathogenesis of spontaneous remission of acute myeloid leukemia (AML).

**Methods:**

We retrospectively analyzed the clinical data of a patient with spontaneous remission of AML, MLL-AF9 rearrangement, and abnormal liver function in the First Affiliated Hospital of Zhengzhou University, and the relevant pieces of literature were summarized.

**Results:**

The patient experienced lung infection, fever, and liver dysfunction and was treated with anti-infection and blood transfusion. After complete response (CR), the patient remained in CR with mild, indirect bilirubin elevation at 35 months of follow-up. Additionally, 56 cases of adult AML (non-acute promyelocytic leukemia) were reported in the literature from 1990 to June 2021. The cases were checked by bone marrow aspiration, and our patients were summarized and analyzed. Furthermore, 57 patients, including 37 males and 20 females, with a median age of 51 (20–83) years and a median remission time of five months; 52 patients achieved complete remission. In addition, there were five cases with long-term remission and a chromosomal record, with no recurrence so far, three with normal karyotype and two with t(9;11) (q21;q23).

**Conclusion:**

The spontaneous remission of leukemia is rare and may be related to immunosuppression and genes.

急性髓系白血病（AML）自发缓解（SR）非常罕见，通常持续时间较短，但有长期缓解的报道。SR的机制可能与全身严重感染、输注血制品、激素水平、基因相关。我们报道一例伴有MLL-AF9基因重排AML患者，该患者出现了血液学和遗传学的完全缓解（CR），同时伴有肺部感染和肝功能异常的痊愈。

## 病例资料

患者，男，48岁，因“间断发热7 d”入住我院。入院前无明显诱因出现发热，多于午后发生，最高体温38.5 °C。于当地医院查血常规：WBC 2.4×10^9^/L，HGB 128 g/L，PLT 66×10^9^/L，中性粒细胞绝对计数（ANC）1.1×10^9^/L。肺部CT：两肺纹理加重；行骨髓细胞形态学、流式细胞术、分子生物学、染色体检查诊断为AML-M_5_，遂转诊至我院。既往史：10个月前于当地医院诊断为“心肌梗死”，植入支架1枚，现口服阿司匹林、氯吡格雷、阿托伐他汀。有“高血压”病史18年，口服缬沙坦控制。入我院后急查血常规（2018年8月28日）：WBC 6.30×10^9^/L，ANC 2.25×10^9^/L，HGB 100.0 g/L，PLT 17×10^9^/L；外周血细胞形态分析：未分类幼稚细胞22％。生化：肌酐158 µmol/L，肾小球滤过率43.908 ml·min^−1^·1.73 m^−2^；丙氨酸转氨酶215 U/L，天冬氨酸转氨酶141 U/L，总胆红素87.50 µmol/L，直接胆红素59.03 µmol/L，间接胆红素28.5 µmol/L；乳酸脱氢酶902 U/L，降钙素原1.610 µg/L，C反应蛋白128.00 mg/L。血培养5 d无细菌生长。腹部超声：肝大并弥漫性回声改变，胆囊壁水肿，右肾体积变小并弥漫性回声改变；心脏超声：左室约58 mm，轻度增大，左室前壁节段性搏动异常（考虑心肌梗死后改变），左室舒张功能下降。我院复查骨髓（图1A）：原始幼稚单核细胞占51.6％。我院读取当地医院骨髓涂片结果（图1B）：原始幼稚单核细胞占58％，过氧化物酶染色：阴性，被NaF抑制。流式细胞术检测：41.31％细胞（占有核细胞）表达CD33、HLA-DR、CD56、CD4、CD64、CD38，不表达cMPO、CD34、CD14、CD117、CD11b、CD13、CD19、CD20、CD10、CD22、TdT、CD2、CD3、CD7、CD8、CD5、CD1a，为恶性幼稚单核细胞。常规融合基因检测：MLL-AF9（+）。基因突变检测：KPAS（+）、PTPN11（+）；染色体核型：t（9;11）（q22；q23）[20]（图2A）。结合以上结果诊断为AML-M_5_。

**图1:**
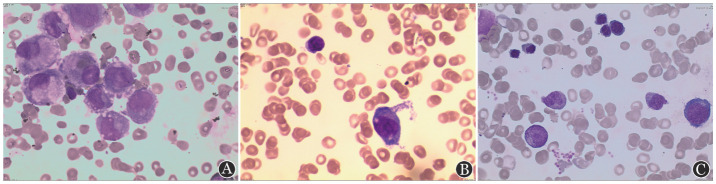
患者初诊时及自发缓解后骨髓象（×400） A：我院初诊时骨髓象可见大量原始幼稚单核细胞；B：当地医院检测骨髓象可见大量原始幼稚单核细胞；C：自发缓解后骨髓象可见各期细胞存在，成熟受阻表型消失

**图2:**
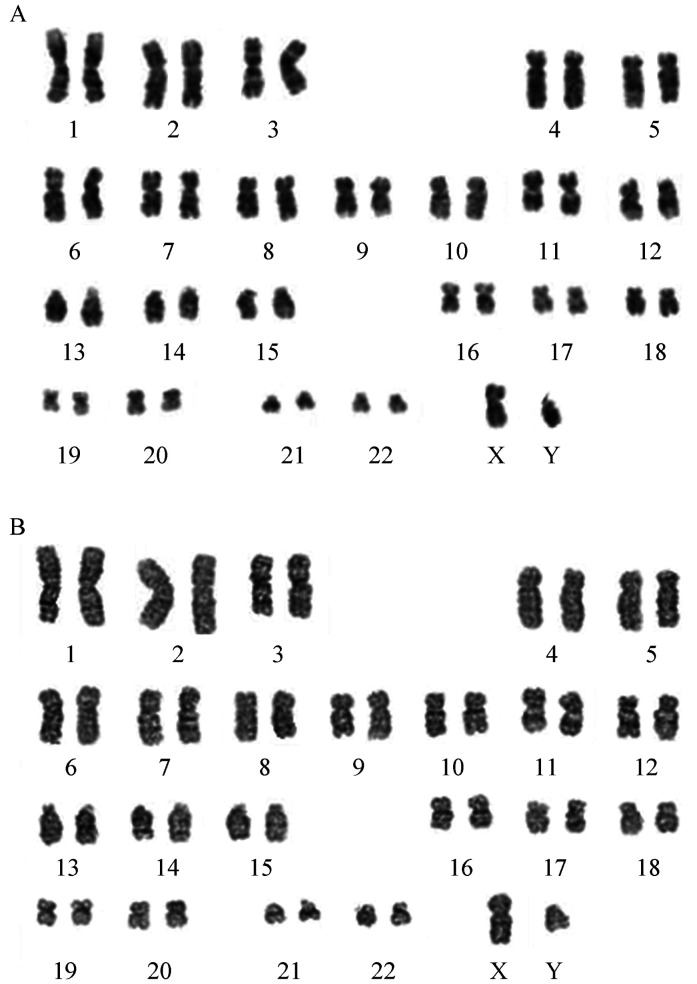
患者初诊时及自发缓解后染色体核型 A：患者在我院初诊时染色体核型分析显示存在t（9;11）（q22；q23）；B：患者自发缓解后染色体分析显示正常染色体核型

患者入院后持续高热（39.0 °C左右），肺部感染，肝功能异常，结合心肌梗死病史，暂缓化疗，给予“比阿培南+替考拉宁+泊沙康唑”抗感染；输注血液制品支持治疗（期间共给予9个治疗量血小板，400 ml冰冻血浆，4 U悬浮红细胞）；对症给予保护肝脏的药物。期间多次复查C反应蛋白和降钙素原，均逐渐下降，C反应蛋白在1个月内由128.00 mg/L降至1.38 mg/L，降钙素原由1.610 µg/L降至0.058 µg/L。入院第28天，患者体温恢复正常，未再发热，肝功能恢复。自患病以来，共发热35 d，热型为弛张热。入院第28天，复查血常规：WBC 1.9×10^9^/L，HGB 74 g/L，PLT 531×10^9^/L；患者血小板恢复，遂复查骨髓（图1C）：原始幼稚单核细胞占1.2％；融合基因MLL-AF9阴性（PCR法检测），基因突变检测：TIN（+）；染色体核型正常（图2B），评估AML CR。入院第36天，体温正常1周，复查血常规：WBC 3.4×10^9^/L，HGB 90 g/L，PLT 376×10^9^/L，办理出院。末次随访时间为2021年7月20日，持续CR约35个月，复查血常规仍在正常范围；复查间接胆红素19.50 µmol/L。

## 讨论及文献复习

肿瘤自发缓解最早由Cole等[1]提出，定义为肿瘤在未经特异性治疗或者靶向治疗后，部分消失或者完全消失。AML SR罕见，既往常被认为是AML误诊。AML SR可发生在婴幼儿时期[2]，也可发生在老年[3]；既可发生于骨髓增生异常综合征[4]，也可发生于继发性AML[5]；既可发生于预后良好细胞遗传学亚组[6]，也可以发生在预后不良细胞遗传学亚组[7]。大多数患者伴有发热、感染；有学者认为输血、终止妊娠与AML SR相关[8]。报道显示多数婴幼儿白血病SR与11q23-MLL相关[9]。我们汇总了1990年至2021年6月30日文献报道的SR成人AML，以“acute myeloid leukemia”或“acute myelogenous leukemia”或“acute myeloblastic leukemia”或“acute leukemia”联合“spontaneous remission”或“spontaneous regression”，检索了PubMed数据库；以“急性白血病”或“急性髓系白血病”联合“自发缓解”检索了“中国知网”“万方数据库”；排除儿童急性白血病、急性早幼粒细胞白血病、急性淋巴细胞白血病、混合表型急性白血病或不明确谱系的病例及初诊和缓解时未行骨髓细胞形态学检查者，加上本例共计57例（表1）。对以上57例患者进行统计分析。

**表1:** 既往报道急性髓系白血病自发缓解患者总结

报道时间及来源	年龄（岁）	性别	FAB分型	骨髓原始细胞（％）	初诊WBC（×10^9^/L）	染色体核型	感染类型	发热	输血	缓解持续时间（月）	复发
本例	48	男	M_5_	58	2.4	t(9;11)(p22;q23)	肺部感染	+	+	35	−
2021[10]	78	男	M_4_	46.5	2.98	NA	NA	NA	−	4	+
2020[5]	66	男	M_2_	35	NA(N:0.5)	正常	未感染	−	−	10.5	+
2020[11]	40	男	NA	75	0.2	正常	髋关节金黄色葡萄球菌感染	+	+	14	−
2020[12]	58	男	NA	45	2.2	正常	急性结肠炎	+	+	24	−
2019[13]	67	男	M_5_	55.5	46	复杂核型^a^	NA	+	+	0.7	+
2019[14]	59	女	M_0_	25	1.3	47, XX, t(4;12）,+mar	手足口病	NA	−	18	+
2019[15]	72	男	NA	85	8	正常	NA	NA	+	12	+
2017[16]	51	男	M_5_	75	NA(N: 0.5)	复杂核型^b^	未感染	+	−	2	+
2017[17]	49	女	M_5_	52	74.9	46, XX, t(8;16)(p11;p13)	未感染	+	−	4	−
2017[18]	53	男	M_4_	75	0.4	正常	肺炎克雷伯杆菌菌血症	+	+	18	−
2016[19]	30	男	M_5_	71.5	1.6	46, XY, t(9;11)(p22;q23)	肺部感染	+	−	2	+
2015[20]	24	女	M_5_	58	2	NA	NA	+	−	2	+
2015[20]	33	男	M_5_	24.5	20	正常	脓毒血症，肺曲霉菌	+	−	1.5	+
2015[20]	74	女	M_5_	55	21	正常	肺炎	+	−	2	+
2015[21]	48	男	M_2_	26.5	0.8	t(10;11)(q21;q23)	肺部曲霉菌感染、肝脏感染	+	NA	0.6	−
2014[22]	77	男	NA	82	2.3	48, XY,+13,+21/46, XY	未感染	+	+	7	+
2014[23]	35	男	M_4_	NA	98.8	NA	未感染	+	+	1.5	+
2013[6]	31	男	M_2_	42	15.3	t(8;21), idem, del(9)	沙雷菌肺炎	+	+	2	+
2013[6]	34	女	MS	0	56.5	正常	NA	+	+	2	+
2012[24]	42	男	M_5_	92	0.5	正常	肺炎	+	NA	27	+
2012[25]	21	男	M_2_	81	0.9	NA	肺部感染	+	−	1	+
2004，2012[26]–[27]	61	男	M_5_	90	0.9	t(9;11)(q21;q23)	肺炎，脓毒血症	+	−	120	−
2011[28]	75	男	NA	90	0.4	+8	肺炎	+	+	5	+
2011[29]	31	女	M_5_	86	1.69	NA	未感染	+	+	NA	NA
2009[30]	63	男	M_2_	34	1.6	del(6)(q21)	未感染	+	+	5	+
2008[31]	66	女	M_4_	43	42.7	+8	肺念珠菌	+	+	29	+
2008[31]	72	女	M_5_	53	NA	NA	NA	NA	NA	5	+
2008[31]	46	男	M_5_	93	0.9	+8	肝脓肿	+	−	2	+
2008[32]	42	女	NA	55	1	复杂核型^c^	上呼吸道感染	+	+	8	−
2007[33]	29	男	M_2_	NA	12	t(8;21),–Y	肺炎	+	+	6	+
2007[33]	28	男	M_5_	NA	1.3	正常	脓毒血症：β-溶血性链球菌	+	+	1	+
2007[34]	83	女	M_5_	60	43.6	+8	尿路感染	+	+	3	+
2007[35]	47	男	M_5_	80	7.4	正常	梭状芽孢杆菌脓毒血症	+	+	4	+
2006[36]	64	男	M_4_	35	22	NA	未感染	+	−	48	+
2005[37]	67	女	NA	24	1.6	正常	NA	NA	+	20	+
2004[38]	31	男	M_5_	95	1.2	正常	G组链球菌菌血症	+	−	2	+
2004[7]	72	男	M_0_	100	2.1	复杂核型^d^	葡萄球菌肺炎、念珠菌	+	+	5	+
2003[39]	34	女	MS	72	56.5	正常	NA	+	+	0.5	+
2001[40]	60	女	M_1_	96	>30	正常	肺曲霉菌	+	+	3	+
2001[41]	71	女	M_2_	49	21	复杂核型^e^	肺炎	+	+	4	−
1998[42]	21	男	M_2_	78	19	t（8;21）	NA	+	+	60	+
1998[42]	32	男	M_2_	76	15.3	NA	咽部感染	+	−	84	−
1996[3]	83	男	M_2_	65	3.6	t(8;21)(q22;q22), del(7)(q22)	肺炎	+	+	1	+
1996[3]	64	男	M_5_	40	44	NA	肠球菌肺炎	+	+	14	−
1995[43]	74	女	M_5_	90	NA	复杂核型^f^	NA	NA	NA	7	+
1994[44]	54	男	M_2_	61.6	0.4	+8	肺炎	NA	NA	NA	+
1994[45]	56	男	M_1_	65	1.1	复杂核型^g^	肺和肝脏结核菌感染	NA	+	34	+
1994[45]	48	男	M_5_	44	5.1	t(8;21),−Y	革兰阴性菌、假丝酵母菌	NA	+	36	+
1994[45]	41	女	M_5_	60	28	正常	未感染	+	+	14	+
1994[45]	54	女	M_4_	45	15	正常	革兰阴性杆菌败血症	NA	+	3	+
1994[46]	49	女	M_5_	80	1	NA	剖腹探查，黏膜溃疡	+	NA	6	+
1993[47]	32	男	M_2_	48	70	NA	疟原虫感染，左下肢感染	+	NA	5	+
1993[48]	72	女	M_0_	64	1.4	三倍体	表皮葡萄球菌肺炎	+	+	5	+
1991[49]	20	男	M_5_	92	6.6	NA	疟原虫感染	+	+	3	+
1991[50]	58	男	M_4_	85	118	NA	卡氏肺孢子虫肺炎	+	+	6	+
1990[51]	47	女	M_5_	NA	NA	+8	未感染	+	−	12	NA

注：MS：髓系肉瘤；NA：无法从原文中获取数据；N：中性粒细胞；+：是；−：否。a：48, XY,+8, inv(9)(p12q13),+18；b：45, XY, t(3;3)(q21;q26), der(17), t(17;21)(p11.2;q11.2)；c：48, XX, del(3)(q21),+6, t(11;15)(q23;q15), +21；d：48, XY, del (6) (p22−pter), +13, +14；e：45, XX, add(4)(q31), t(8;21)(q22;q22), add(18)(q21),+mar；f：46, XX, t(9;11)(p22;q23）/52, XX,+3,+8,+8,+14,+19, t(9;11)(p22;q23）；g：50, XXY,+4,+8,+14.+t(2lq: 22q),−21,−22

由于样本量较小且呈偏态分布，我们采用中位数来描述集中趋势。病例特点如下：男37例，女20例；诊断时中位年龄51（20～83）岁；中位WBC 2.98（0.2～118.0）×10^9^/L；中位骨髓原始细胞61.6％（24％～100％）；中位缓解时间5个月；共50例患者具有FAB分型记录：其中M_5_ 24例，M_2_ 12例，M_4_7例，M_0_ 3例，M_1_ 2例，髓系肉瘤（MS）2例；所有患者中M_4_和M_5_占比达62.0％。57例患者中，有7例为继发AML。染色体核型分析：13例未获得染色体核型；44例染色体核型如下：正常核型15例；＋8异常6例；单纯t（8;21）1例；t（8;21）伴有其他异常5例；t（9;11）（MLL）3例；t（10;11）（MLL）1例；复杂染色体核型7例，其中1例含t（11;15）（MLL），1例含t（9;11）（MLL），1例含t（8;21）；其他7例。根据2017年AML成人国际专家小组ELN建议，按照细胞遗传学危险度分层，低危1例（0.2％），中危31例（70.5％），高危12例（27.3％）。感染情况：47例有感染相关资料患者中37例（78.7％）发生明确感染，10例（21.3％）未发生感染。最常见感染类型：肺炎22例，肺炎患者中2例伴有肝脏感染；菌血症8例，其中4例患者脓毒血症可能是因为肺部感染扩散，因为患者伴有重症肺炎；其他感染10例，包含疟原虫感染2例，髋关节感染、急性结肠炎、手足口病、肝脓肿、上呼吸道感染、尿路感染、咽部感染、剖腹探查手术后各1例。发热症状：47例有发热情况资料的患者中37例（78.7％）出现发热；10例无发热。输血情况：34例（58.6％）输血，16例（28.0％）未输血，7例（12.3％）患者输血情况未知；获得持续CR患者中输血13例，未输血5例。对于缓解深度进行分析，52例患者获得了CR，5例获得了部分缓解。CR患者占比高，可能由于AML获得自发CR病例更为典型，所以个案报道相对较多。复发44例，中位缓解时间5.0个月，平均缓解时间9.56个月；有1例缓解长达5年后复发[42]；有1例缓解时间≥10年未复发[27]。

我们定义缓解时间大于1年为长期缓解，其中18例获得了长期缓解，男13例，女5例；中位年龄54.5（21～72岁）；中位骨髓原始细胞60％（24％～92％）；中位WBC 2.4（0.2～42.7）×10^9^/L；具有FAB分型者共有14例，其中M_5_ 7例，M_4_ 3例，M_2_ 2例，M_0_与M_1_各1例；其中有2例为继发白血病，1例继发于慢性粒-单核细胞白血病，1例继发于骨髓增生异常综合征；可获取染色体核型者15例，危险度分层：低危1例，中危13例，高危1例；可获取感染情况者共15例，感染12例（80.0％），未感染3例（20％）；可获取发热情况者共13例：发热10例（77.0％），不发热3例（23.1％）；可获取输血与否者共17例，输血12例（70.6％），未输血5例（29.4％）。缓解深度：CR 16例，部分缓解2例；中位缓解时间为22个月。复发10例，未复发7例，失访1例。长期缓解且没有复发的患者共7例，其中1例缓解长达10年，与本例患者具有相同细胞遗传学特征：t（9;11）（q21;q23），且本例患者至今仍在随访中，CR时间已达35个月，未复发。

AML SR罕见，随着化疗的广泛应用，更进一步降低了此种现象的发生。选择支持治疗的患者，大多数伴有严重感染，或者体力状态较差。我们总结的57例患者，长期缓解病例共计18例；44例最终复发，推测患者机体免疫和白血病细胞之间处于动态平衡，在遇到外界感染等打破这种平衡时，白血病有可能复发[14]。大多数AML起病时，皮肤和微小伤口的脓疱或者其他轻微的化脓性感染较为常见，严重的感染，如鼻窦炎、肺炎、肾盂肾炎、脑膜炎等在起病时较为少见。随着化疗后粒细胞缺乏，严重的感染才变得频繁。许多患者诊断时伴随发热，但发热时不一定存在感染[52]。我们统计的57例患者中，感染与发热患者均为37例，但是感染与发热并不完全重合。AML SR患者中，最常见的感染是细菌感染，也有真菌感染和病毒感染。发热、感染过程中促炎细胞因子的释放可导致肿瘤细胞热休克蛋白（HSP）的表达增加，HSP被认为在肿瘤的SR中起重要作用[53]；目前关于HSP清除肿瘤细胞的研究主要集中在实体瘤。局部热疗在实体瘤的治疗中起到一定作用，但尚无证据表明热疗能对伴有转移的实体瘤起作用，局部热疗尚不足以激活全身的免疫系统[54]；由此推测AML SR较实体瘤的SR更为罕见，可能与全身免疫系统激活程度有关。严重感染时肿瘤坏死因子（TNF）及白细胞介素-2（IL-2）水平显著升高[48]，这些细胞因子的多重复杂作用，导致白细胞破坏性血管炎，证明感染时免疫系统激活在AML SR中发挥作用。此外，感染诱导G-CSF的产生，G-CSF可增加效应细胞毒性，抑制白血病细胞[35]。用化脓性链球菌提取物、卡介苗、内毒素、双特异性T细胞抗体（BiTE）、表达嵌合抗原受体（CAR）的T细胞等方法进行免疫治疗，AML患者的缓解率显著提高，生存时间显著延长[23]。免疫系统对白血病细胞生长的内源性抑制可能并不局限于SR的患者。有令人信服的证据表明，异基因造血干细胞移植后的微小残留病是由移植物抗白血病反应控制的；类似的机制也可能涉及自体移植甚至常规化疗后的微小残留病。

本例患者具有t（9;11）（q21;q23）异常，与Müller等[26]报道的1例缓解长达10年的患者，具有相同的细胞遗传学异常。AML SR发生在细胞遗传学预后良好组，似乎更容易被人接受。在57例AML SR患者中，44例患者有细胞遗传学资料，中低危组占77.2％，伴不良细胞遗传学异常患者占22.7％。在大约10％的患唐氏综合征的婴儿中，一过性骨髓增殖性疾病（TAM）可在出生时或出生不久后发生，约80％患儿可在数周至数月内消失[55]。这种比较典型的情况说明了细胞遗传学对AML预后的重要性。AML SR是否与特定细胞遗传学相关，有待进一步研究。Rady等[56]报道1例AML患者在诱导治疗后出现了异常克隆造血，表现为der（6）t（6;13），诊断为治疗相关性髓系肿瘤（t-MN），之后患者未经过任何相关治疗，在诊断后10年，der（6）t（6;13）经历了克隆增多到退化至无法检测，说明了异常克隆造血失败；亦有报道自发PNH克隆消失[57]。均提示不同的基因导致的异常克隆生存能力不一，基因对白血病的预后有着极大的预测价值；t（9;11）（q21;q23）异常/MLL-AF9基因重排的AML是否更容易发生异常克隆造血的失败，有待进一步研究证实。

输血可能与白血病SR有关，供者血液中含有的活性物质如细胞因子、NK细胞和T细胞，具有潜在的抗白血病活性。供者来源细胞毒性T细胞及B细胞可在患者体内增殖并发生免疫反应[58]；临床上，许多白血病患者经历过感染和（或）输血，但SR的患者极少，提示SR是多种因素共同作用的结果。此外，我们常规使用去白细胞或者辐照血制品，也降低了AML SR的发生[59]。妊娠并发AML SR仅有4例报道[29]，其中1例28岁女性，产后AML很快缓解，3个月后，乳房有大量白血病细胞浸润，疾病复发，终止妊娠后缓解和复发的初始部位主要在乳房，强烈提示白血病细胞依赖激素，并支持终止妊娠时激素变化导致SR的观点。AML患者雌激素受体（ER）异常高表达已被证实[60]。推测妊娠伴随AML的患者有激素反应性AML细胞，在终止妊娠时激素变化导致细胞退化，当激素环境恢复到最佳状态时，在激素敏感的组织（乳房）中迅速增殖。

本例患者获得了长期缓解，患病期间伴有间接胆红素异常升高，且在缓解期间，随访仍有轻度的间接胆红素升高。胆红素具有抗氧化和抗炎的特性[61]，胆红素对吞噬细胞的抗原提呈功能起负向调节作用，从而导致免疫活性细胞抗原刺激的减少甚至消除；接近生理血清浓度的间接胆红素可以影响固有免疫中补体生理[62]，是否与患者的长期缓解相关，有待进一步研究。
